# The purpose and effectiveness of doping testing in sport

**DOI:** 10.3389/fspor.2024.1386539

**Published:** 2024-05-13

**Authors:** Fredrik Lauritzen, Anders Solheim

**Affiliations:** ^1^ Science and Medicine, Anti-Doping Norway, Oslo, Norway; ^2^ Anti-Doping Norway, Oslo, Norway

**Keywords:** anti-doping, doping control, athlete, clean sport, AAF, ADRV, World Anti-Doping Agency, WADA

## Abstract

Maintaining an effective testing program is critical to the success and credibility of the anti-doping movement. However, a low detection ratio compared to the assumed real prevalence of sport doping has led some to question and criticize the effectiveness of the current testing system. In this perspective article, we review the results of the global testing program, discuss the purpose of testing, and compare benefits and limitations of performance indicators commonly used to evaluate testing efforts. We suggest that an effective testing program should distinguish between preventive testing and testing aimed at detecting the use of prohibited substances and prohibited methods. In case of preventive testing, the volume of the test program in terms of number of samples, tests and analyses is likely to be positively related to the extent of the deterrent effect achieved. However, there is a lack of literature on how the deterrent effect works in the practical context of doping testing. If the primary goal is to detect doping, the testing must be risk- and intelligence-based, and quality in test planning is more important than quantity in sample collection. The detection ratio can be a useful tool for evaluating the effectiveness of doping testing, but for the calculation one should take into account the number of athletes tested and not just the number of collected samples, as the former would provide a more precise measure of the tests’ ability to detect doping among athletes.

## Introduction

For decades, athletes have used performance-enhancing substances and methods to improve athletic performance and gain a competitive edge. Mainly to protect the health of athletes from potentially harmful doping practices, the first significant anti-doping initiatives were introduced in the 1970s (1). In response to growing concerns, the World Anti-Doping Agency (WADA) was established in 1999 by the Sport Movement and Governments of the world to co-ordinate the global fight against doping and to protect athletes’ fundamental right to participate in doping-free sport, taking over the responsibility for anti-doping from the International Olympic Committee Medical Commission. A few years later, WADA released the first edition of the World Anti-Doping Code (WADC). The WADC was quickly adopted and enforced by international sport organisations and National Anti-Doping Organisations (NADOs) worldwide, and acknowledged by governments through the UNESCO convention (2). Today, the WADC together with eight mandatory International Standards and Technical Documents and 12 non-mandatory Guidelines constitutes the World Anti-Doping Program, which seeks to harmonize anti-doping policies, rules and regulations across sports and public authorities (3).

Since the establishment of WADA, anti-doping has become increasingly multi-disciplinary. To prevent and detect doping, modern anti-doping programs include disciplines such as analytical chemistry, education, forensic science, pharmacology, physiology, psychology, and law. However, despite the increasing complexity of the World Anti-Doping Program, the collection and analysis of biological samples from athletes accounts for more than half of the global anti-doping budget (4) making it the main activity for most anti-doping organisations (ADOs). Providing effective and cost-efficient testing programs is therefore essential for the success and credibility of the anti-doping movement.

The word e*ffective* is used several times in the WADC. For example, the first part of the Code, which describes the purpose, scope and organisation of the World Anti-Doping Program and the WADC, states in relation to detection that “an *effective* testing and investigations system not only enhances a deterrent *effect*, but also is *effective* in protecting clean athletes and the spirit of sport by catching those committing anti-doping rule violations, while also helping to disrupt anyone engaged in doping behaviour” [p. 9, (3)]. The current International Standard for Testing and Investigations provide several recommendations for conducting effective testing (5), however, it is still somewhat unclear how it can be measured and evaluated.

Doping testing practices have not been immune to criticism. Most notably, a significantly lower detection ratio of positive samples compared to the assumed true prevalence of athletes doping has led some to question and criticise the effectiveness of the doping efforts (6, 7), suggested that current practices are unfit to detect doping (8), and that anti-doping authorities are more concerned with the number of samples collected than on exposing doping (9).

In this article, we critically discuss the concept of effectiveness in the context of doping testing in sport, the purposes of testing, as well as the validity of the figures and performance indicators that are often used to measure and evaluate its success. We argue that there is a need for more precise and harmonized indicators to better measure the doping test regimes’ ability to detect and deter doping, and that implementation of more intelligent and data-driven testing by ADOs may increase the quality and effectiveness of the global testing program.

## Determining the success of doping testing—does testing numbers count?

The unofficial parameter used by anti-doping practitioners to measure whether adequate measures are taken to combat doping has traditionally been the number of samples or tests carried out by a given ADO or within a specific sport or country. In general, the notion has been that the more you test, the better program you have. However, global test statistics from the last two decades suggest that increased testing has not translated into a corresponding increase in the proportion of positive tests (6, 10). According to the WADA Anti-doping Testing Figures report, which was first presented in its current form in 2012, there was a 35% increase in the total number of annual samples reported into WADA's Anti-Doping Administration and Management System (ADAMS) from 2012 (206 391 samples) to 2019 (278 047 samples) (11), after which the Covid-19 pandemic resulted in a widespread suspension or reduction in most anti-doping activities in 2020 (12). Interestingly, the number of samples with a positive finding for a prohibited substance or method, what is referred to as an Adverse Analytical Finding (AAF), only increased 6% in the same period (2,549 to 2,702 AAFs).

Adverse Analytical Findings, however, should not be confused with doping violations, as some AAFs are dismissed for medical or other reasons. More appropriate figures for assessing the success of the global testing efforts in detecting doping can instead be found in the WADA ADRV reports, first released in 2013. An ADRV is defined as a doping case for which a final decision has been rendered and a sanction was imposed against the athlete or athlete support personnel (3). The ADRVs are separated into analytical ADRVs, which are based on AAFs, and non-analytical ADRVs, which are based on other types of rule violations. Statistics on ADRVs may offer several advantages when evaluating testing efforts, however, not all ADRVs are related to intentional doping as some AAFs are caused by inadvertent ingestion of prohibited substance (13), for example through food or dietary supplements (14, 15).

## Calculating the detection ratio

A starting point for evaluating the effectiveness of testing programs is to calculate and assess the detection ratio, which can be done in several ways. Using analytical ADRVs and all samples collected by ADOs worldwide (except samples for the Athlete Biological Passport as these are not for direct detection of prohibited substances or methods) gives a detection ratio of 0.66% for the period 2013–2019 (10 759 analytical ADRVs from 1 640 999 collected samples) (16). In contrast to analytical ADRVs, most types of non-analytical ADRVs are not related to testing and should rightfully not be included when evaluating effects of doping testing. There are certain exceptions, such as (a) Use or attempted use of a prohibited substance or method, (b) Evading, refusing, or failing to submit to sample collection, and (c) Tampering with any part of a doping control, all of which are potentially related to testing (3). Adding these non-analytical ADRVs to the analytical ADRVs result in a slightly higher ADRV-to-sample ratio for the period 2013–2019, which would still be well under 1%.

## The prevalence of athletes doping

Does an ADRV-to-sample ratio of less than one percent reflect that the current testing strategy is successful, or rather that it has severe limitations in exposing cheaters? For any meaningful evaluation of the detection ratio to take place it should be compared with the relative number of athletes doping. Unfortunately, the true prevalence of sport doping has been challenging to estimate with any degree of certainty (17). A recent evidence synthesis report a doping prevalence in competitive sport between 0% and 73% (13). The high variation between studies is not surprising considering the different methodological approaches used to measure prevalence (13), and given the varying benefits of doping across sports, differences in sporting cultures, athletes’ knowledge of anti-doping rules etc. (18, 19). The importance of reliable methods for adequate assessment of doping prevalence has been acknowledged by WADA, which has established a Prevalence Working Group to provide more accurate numbers.

## The purpose of doping testing—detection vs. deterrence

Doping testing is not exclusively undertaken to obtain analytical evidence of the use of prohibited substances or methods in the form of positive samples. Although the analytical methods used to analyse biological samples from athletes are continuously improving [e.g., (20, 21)], testing in itself continue to have several limitations in exposing doping, including but not limited to a short window of detection and low test sensitivity for certain substances, and high predictability of testing (8). In view of these shortcomings, it has been suggested that it is necessary to carry out 16–50 tests per athlete per year to uncover all doping cases (8). In addition to being ethically questionable, the cost of such a hypothetical program would not be economically viable. Considering the difficulties of the detection-based approach, it has thus been argued that the global testing program is mainly dependent on *deterring* athletes from making the decision to dope by risk of detection and severe sanctions (22).

According to the theory of deterrence, if athletes perceive that there is a high probability of detection and they consider the consequences to be severe, they are less likely to break the rules (23, 24). For sanctions following positive doping tests to provide credible threats and act as a deterrent to doping practices, it is estimated that the perceived certainty of punishment must be 30% or higher (25). According to the deterrence theory, the more frequent athletes are tested, and the more samples that are collected, the greater certainty of punishment and thus deterrence is achieved. This effect is likely to apply up to a certain point, where more testing will not result in further increases in deterrence. In line with this, it has been shown that athletes with personal experience with testing and who are tested regularly are more likely to experience a deterrent effect (26). Conversely, athletes who lack confidence in the system and perceive that doping controls are unable to detect doping do not believe that the current testing program is a strong deterrent (27). Another key component of deterrence is celerity, i.e., that the sanction are imposed swiftly after the offense for the transgressor to connect the violation with the punishment (25). How long the Result management process in a doping case lasts before a sanction is imposed will thus affect the athlete's perception of the deterrent effect of testing.

A possible explanation for the reduction in the detection ratio in the global testing program from 2013 to 2019 is that the annual increases in sample collection have resulted in an enhanced deterrent effect among athletes, resulting in fewer relative ADRVs. Such a scenario is in line with how the theory of deterrence can be expected to work in practice. It is not surprising that athletes who are subjected to regular random doping testing experience the risk of being caught so high that they refrain from using prohibited substances.

More research should be carried out to gain a better understanding of how the deterrent effect takes place in the practical context of doping control. Establishing the threshold for when a satisfactory level of deterrence is reached will be of great interest to ADOs and could contribute to more efficient use of testing resources. There is no reason to continue testing an athlete 15 times a year unless there is specific confidential source information indicating doping use, if future research suggests that a satisfactory level of deterrence is achieved with, say, seven randomly assigned annual tests.

## Discussion and recommendations for improving testing effectiveness

Several requirements and recommendations has been made in the last decade with the goal to make testing more targeted and effective [e.g., (28)]. Nevertheless, ten years after that the lack of effectiveness in the testing program in sport was discussed by WADA (6), ADOs are still struggling to detect doping among athletes. In view of the admittedly low detection rate and to meet the criticism that anti-doping has become a “numbers game”, ADOs should consider taking several measures to increase the quality and effectiveness of their testing programs.

### Prioritize quality vs. quantity in testing when the goal is to detect doping

Insufficient funding has been used to explain the lack of effectiveness of doping testing (6). Indeed, doping controls are expensive and all ADOs operate with limited budgets. However, as we have previously discussed, there is no automaticity that administering more doping testing will result in a higher number of positive samples either in absolute or relative terms (6, 9, 29, 30). Instead of increasing the budget to accommodate increased sample collection and analysis, ADOs should improve the risk assessment process for better target testing. To put it simply, when aiming to detect doping, test smarter, not more.

To gain more knowledge about high-risk athletes and sports in a respective country or region, ADOs should examine their own historic test and ADRV statistics. Sharing of practices on how ADOs use intelligence in the test planning process, and how it affects the detection rates should be encouraged and will contribute to a more data-driven approach to test planning in the anti-doping community.

### Invest in building intelligence capabilities

The importance of information-based testing and the use of forensic methods and intelligence (28, 29, 31), as well as cross sectional cooperation (32) to uncover both analytical and non-analytical rule violations has been increasingly promoted in the last decade. ADOs should therefore invest in human resources which may increase their capability and capacity to gather and use intelligence in test planning and set up a system that allows for the collection and processing of information on possible rule violations. Whistle blowing/tip offs, sport performance data, social media activity, athlete biological profiles, previous testing records, whereabouts information and information from law enforcement are all potential sources of relevant information which could be used to increase the quality of the test planning process. To free up resources to increase investments in intelligence capacity, ADOs can consider reducing some testing in low-risk sports and of athletes with a long and clean record and where there are not indications of rule violations.

### Distinguish between tests for deterrence and for detection

In theory, doping controls have both a deterrent effect and the potential to detect doping (22). In practice, many tests are mainly preventive in the sense that there exist no suspicion or specific information about potential doping use by the tested athlete. The main purpose of these test is to *deter* the athlete from future use of a prohibited substance or method. Separating the samples collected for preventive purposes from those collected with the aim of detecting doping when calculating the detection ratio would give a more precise picture of the actual ability of doping tests to detect doping.

### Improved reporting of test statistics

The annual WADA reports which present global testing numbers and analytical findings represent the best available source of statistics for evaluating global testing efforts. As previously explained, the reports provide various figures that could potentially be used for this purpose, but their current format does not make the content easily accessible to outside observers. It has therefore been suggested to reform WADAs reporting system in order to make it easier to evaluate the impact, efficiency and proportionality of the policies and programmes in place (33). Improved reporting practices can also in itself contribute to countering doping, in addition to strengthen individual and public trust in the anti-doping system (33). Gleaves et al. (13) have recently proposed several recommendations for reporting guidelines relating to measurements of doping behaviour which are also relevant for evaluation of testing efforts. Among these the most significant is the importance of also presenting the number of athletes tested in a given period, sport, or country and not only the number of samples collected. Most athletes are tested several times per year. For example, it is not unusual for high-profile athletes participating in sports that are considered to have a high risk for doping, such as disciplines that require high levels and degrees of specialisation in endurance, strength, or power, to provide ten or more doping samples annually. If twenty athletes together provide two hundred samples over the course of a year, of which one sample comes back positive for a prohibited substance, this would, with normal calculations give a detection ratio of 0.5%. However, it is equally true that five percent of the athletes who were tested returned a positive sample, which gives a completely different conclusion on whether the testing of these twenty athletes was successful in detecting doping or not. By calculating the proportion of ADRVs per number of athletes rather the per number of samples, the detection ratio will probably be closer to the real doping prevalence.

Lastly, the ADRVs are currently grouped as analytical or non-analytical, but neither category fully encompasses the ADRVs related to doping testing. To evaluate the outcome of testing, a new category that includes all test-related ADRVs would be useful.

## Conclusions

Consistent and adequate funding is necessary to run a high-quality anti-doping program. However, more funding will not automatically improve the output of testing programs if the resources are not used wisely. Anti-doping organizations’ intelligence and investigation capability and capacity should be strengthened and considered as an integral part of testing operations. If necessary, collecting fewer samples can free up financial resources to enable improved target testing of at-risk athletes and sport environments. Performing high quality risk assessments on both the individual, team and sport discipline level should be considered as pivotal. More studies should be done to examine the relationship between the volume of samples and the deterrent effect. Reducing the number of samples should, however, not come at the expense of the preventive and deterrent effect of doping testing.

Most athletes want to compete clean and support the various measures imposed on them by sport and ADOs (34). However, there is no automaticity in the fact that this will persist, and some athletes already question the lack of efficiency and equality across sports and countries (34). To maintain the trust of athletes, governments, and other stakeholders in the world of sports, ADOs should take measures to improve testing effectiveness and facilitate the evaluation of their practices through transparent reporting of testing figures and results (33).

Finally, anti-doping is more than sample collection and detection ratios. In this article we have limited the discussion to testing. However, a similar exercise should be done for other areas within anti-doping, such as education, which is now considered a cornerstone of global anti-doping efforts, and an important prevention strategy for a successful fight against doping (35), but where the effect of the majority of the various programs is not well known (36).

## Data Availability

The original contributions presented in the study are included in the article/Supplementary Material, further inquiries can be directed to the corresponding author.
